# *N*,*N*-Dibenzoyl­ferrocenecarboxamide

**DOI:** 10.1107/S1600536811013754

**Published:** 2011-04-16

**Authors:** Mario Cetina, Veronika Kovač, Vladimir Rapić

**Affiliations:** a Department of Applied Chemistry, Faculty of Textile Technology, University of Zagreb, Prilaz baruna Filipovića 28a, HR-10000 Zagreb, Croatia; b Laboratory of Organic Chemistry, Faculty of Food Technology and Biotechnology, University of Zagreb, Pierottijeva 6, HR-10000 Zagreb, Croatia

## Abstract

In the title compound, [Fe(C_5_H_5_)(C_20_H_14_NO_3_)], the cyclo­penta­dienyl rings deviate by 9.3 (2)° from an eclipsed conformation, defined by C—*Cg*_1_—*Cg*_2_—C pseudo-torsion angles ranging from 8.8 (1) to 9.85 (1)°. The coordination at the N atom is slightly pyramidal, as indicated by the angular sum around it of 352.6°. The amide group is inclined at 17.86 (9) and 27.27 (11)° with respect to the aromatic rings. In the crystal, mol­ecules are linked by one C—H⋯O hydrogen bond and one C—H⋯π inter­action into a two-dimensional framework parallel to the *b* axis.

## Related literature

For background to ferrocene amides, see: Kohmoto *et al.* (2008 ▶); Masu *et al.* (2005 ▶, 2006 ▶); Moriuchi *et al.* (1995 ▶, 2000 ▶); Moriuchi & Hirao (2007 ▶). For hydrogen-bond motifs, see: Bernstein *et al.* (1995 ▶). For a description of the Cambridge Structural Database, see: Allen (2002 ▶).
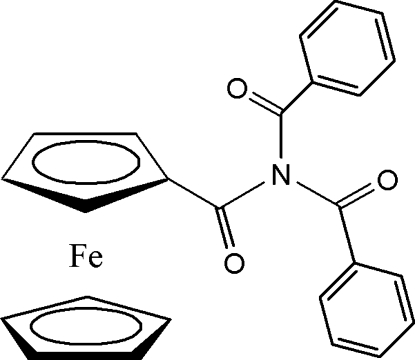


## Experimental

### 

#### Crystal data

[Fe(C_5_H_5_)(C_20_H_14_NO_3_)]
*M* *_r_* = 437.26
Monoclinic, 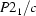

*a* = 10.8699 (4) Å
*b* = 11.3387 (4) Å
*c* = 19.6264 (7) Å
β = 122.133 (2)°
*V* = 2048.42 (13) Å^3^
*Z* = 4
Mo *K*α radiation
μ = 0.76 mm^−1^
*T* = 295 K
0.56 × 0.53 × 0.43 mm

#### Data collection

Oxford Diffraction KM-4/Xcalibur diffractometer with a Sapphire3 detector
Absorption correction: multi-scan (*CrysAlis RED*; Oxford Diffraction, 2009 ▶) *T*_min_ = 0.910, *T*_max_ = 1.000
14704 measured reflections
5867 independent reflections
3472 reflections with *I* > 2σ(*I*)
*R*_int_ = 0.024

#### Refinement

*R*[*F* ^2^ > 2σ(*F* ^2^)] = 0.032
*wR*(*F* ^2^) = 0.068
*S* = 0.93
5867 reflections
271 parameters
H-atom parameters constrained
Δρ_max_ = 0.26 e Å^−3^
Δρ_min_ = −0.24 e Å^−3^

### 

Data collection: *CrysAlis PRO* (Oxford Diffraction, 2009 ▶); cell refinement: *CrysAlis PRO*; data reduction: *CrysAlis PRO*; program(s) used to solve structure: *SHELXS97* (Sheldrick, 2008 ▶); program(s) used to refine structure: *SHELXL97* (Sheldrick, 2008 ▶); molecular graphics: *WinGX* (Farrugia, 1999 ▶); software used to prepare material for publication: *publCIF* (Westrip, 2010 ▶).

## Supplementary Material

Crystal structure: contains datablocks I, global. DOI: 10.1107/S1600536811013754/im2277sup1.cif

Structure factors: contains datablocks I. DOI: 10.1107/S1600536811013754/im2277Isup2.hkl

Additional supplementary materials:  crystallographic information; 3D view; checkCIF report

## Figures and Tables

**Table 1:** Hydrogen-bond geometry (Å, °) *Cg*2 is the centroid of the C6–C10 ring.

*D*—H⋯*A*	*D*—H	H⋯*A*	*D*⋯*A*	*D*—H⋯*A*
C4—H4⋯O1^i^	0.93	2.56	3.241 (2)	131
C15—H15⋯*Cg*2^ii^	0.93	2.97	3.528 (2)	120

Symmetry codes: (i) 
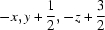
; (ii) 
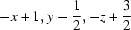
.

**Fig. 1.**
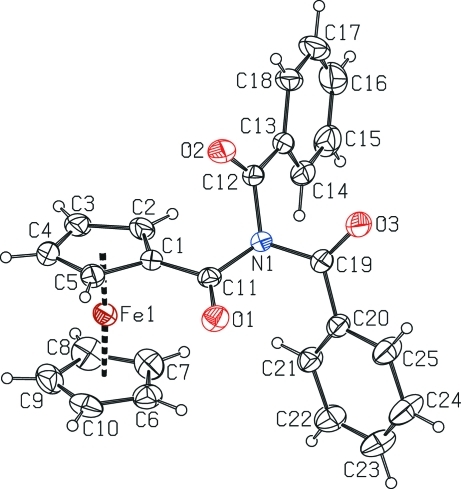
Molecular structure of 1, with the atom-numbering scheme. Displacement ellipsoids for non-hydrogen atoms are drawn at the 30% probability level.

**Fig. 2.**
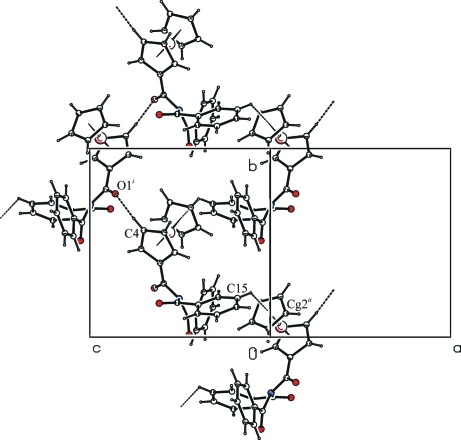
Crystal packing diagram of 1, showing the C–H···O hydrogen bonds and C–H···π interactions as dashed lines. Symmetry codes are given in Table 1.

## supplementary crystallographic information

### Comment

Recently, aromatic foldamers with iminodicarbonyl linkers have been synthesized to investigate their structures in comparison with that of biopolymers, mostly proteins (Masu *et al.* 2005, 2006; Kohmoto *et al.* 2008). Also, considerable interest has been devoted to the synthesis of various imide based ferrocene derivatives in which the redox properties of transition metals permits their potential utilization as electrical materials and catalysts, *e.g.* ferrocenophanes (Moriuchi *et al.*, 1995, 2000) or imide-bridged diferrocene for protonation-controlled regulation of electronic communication (Moriuchi & Hirao, 2007). Considering these previous studies we decided to prepare some new ferrocene containing imides notably due to investigation of their structure. During the synthesis of model substances as a part of our current research on ferrocene foldamers with iminodicarbonyl linkers we have isolated the title compound 1 and its structure is described in this paper.

A survay of Cambridge Structural Database (Allen, 2002) revealed that 1 (Fig. 1) is the first structure with a N–(C=O)_3_ fragment linked to the ferrocenyl moiety. N1 is pyramidal and is displaced by 0.228 (1)Å out of the plane defined by atoms C11/C12/C19. Because of pyramidality, the angular sum around N1 is 352.6°. A somehow close C14–H14···N1 interaction of 2.55 Å seems to be indicative for a intramolecular hydrogen bond forming a five-membered ring that could be described by graph set descriptors as *S*(5) (Bernstein *et al.*, 1995). On the other hand, the C14–H14···N1 angle is very small (101°) and there is no difference in chemical shifts for protons at C14 and C18 in the ^1^H NMR spectrum (7.91 p.p.m., doublet, for 4 protons of the C14, C18, C21, C25), therefore indicating the absence of an intramolecular hydrogen bond at least in solution.

The C11=O1 carbonyl group and C1–C5 ring form an extended π-conjugated system, and therefore C1–C11 bond is shortened. The coplanar arrangement of a carbonyl group attached to the cyclopetadienyl (Cp) ring should allow maximum interaction of two π-systems. The C11=O1 bond is twisted out of the plane of the C1–C5 ring for 18.81 (11)°. The Cp rings deviate 9.3 (2)° from an eclipsed conformation. The value of the C–*Cg*_1_–*Cg*_2_–C pseudo-torsion angles, defined by joining two eclipsing Cp carbon atoms through the ring centroids range from 8.8 (1) to 9.8 (1)°. The Cp rings are almost parallel, with a dihedral angle between the mean planes of the rings of 1.84 (11)°. The amide groups do not lie in the plane of the attached aromatic rings. Thus, the plane of the C13/C12/O2/N1 atoms is inclined to the C13–C18 phenyl ring for 17.86 (9)°, and the plane of the C19/C20/O3/N1 atoms is inclined to the C20–C25 phenyl ring for even 27.27 (11)°. Two phenyl rings are perpendicular with respect to the C1–C5 ring. The corresponding dihedral angles are 85.36 (9) and 88.17 (10)° for C13–C18 and C20–C25 rings, respectively.

Molecules of 1 are self-assembled by C4–H4···O1 hydrogen bonds (Fig. 2; Table 1) into *C*(6) (Bernstein *et al.*, 1995) spirals parallel to the *b* axis. Hydrogen-bonded chains are further weakly linked by one C–H···π interaction, C15–H15···*Cg*2, also parallel to the *b* axis forming a two-dimensional framework.

### Experimental

To a suspension of hexane-washed sodium hydride (60% NaH in mineral oil, 78 mg; 2.027 mmol) in dry tetrahydrofuran (5 ml), a solution of ferrocene amide (188 mg; 0.821 mmol) in dry tetrahydrofuran (5 ml) was dropped and the mixture was heated at reflux for 2 h. After cooling benzoyl chloride (0.13 ml; 1.126 mmol) was added and the reaction mixture was stirred at room temperature overnight. After removing the solvent the remaining residue was dissolved in dichloromethane, washed with water and brine, dried over anhydrous sodium sulfate and evaporated. TLC purification gave 111 mg (41%) orange crystals of *N*-benzoylferrocenecarboxamide 2 and 76 mg (21%) red crystals of the title compound 1. A single-crystal of 1 was grown by slow evaporation from a saturated dichloromethane solution.

### Refinement

All H atoms were included in calculated positions as riding atoms, with *SHELXL97* (Sheldrick, 2008) defaults, *viz*. C–H = 0.93 Å and *U*_iso_(H) = 1.2 *U*_eq_(C).

### Crystal data

**Table:**

[Fe(C_5_H_5_)(C_20_H_14_NO_3_)]	*F*(000) = 904
*M**_r_* = 437.26	*D*_x_ = 1.418 Mg m^−^^3^
Monoclinic, *P*2_1_/*c*	Melting point = 416–418 K
Hall symbol: -P 2ybc	Mo *K*α radiation, λ = 0.71073 Å
*a* = 10.8699 (4) Å	Cell parameters from 5978 reflections
*b* = 11.3387 (4) Å	θ = 4.0–31.8°
*c* = 19.6264 (7) Å	µ = 0.76 mm^−^^1^
β = 122.133 (2)°	*T* = 295 K
*V* = 2048.42 (13) Å^3^	Prism, red
*Z* = 4	0.56 × 0.53 × 0.43 mm

### Data collection

**Table:**

Oxford Diffraction KM-4/Xcalibur diffractometer with a Sapphire3 detector	5867 independent reflections
Radiation source: Enhance (Mo) X-ray Source	3472 reflections with *I* > 2σ(*I*)
graphite	*R*_int_ = 0.024
Detector resolution: 16.3426 pixels mm^-1^	θ_max_ = 30.0°, θ_min_ = 4.1°
ω scans	*h* = −15→14
Absorption correction: multi-scan (*CrysAlis RED*; Oxford Diffraction, 2009)	*k* = −10→15
*T*_min_ = 0.910, *T*_max_ = 1.000	*l* = −27→27
14704 measured reflections	

### Refinement

**Table:**

Refinement on *F*^2^	Primary atom site location: structure-invariant direct methods
Least-squares matrix: full	Secondary atom site location: difference Fourier map
*R*[*F*^2^ > 2σ(*F*^2^)] = 0.032	Hydrogen site location: inferred from neighbouring sites
*wR*(*F*^2^) = 0.068	H-atom parameters constrained
*S* = 0.93	*w* = 1/[σ^2^(*F*_o_^2^) + (0.0306*P*)^2^] where *P* = (*F*_o_^2^ + 2*F*_c_^2^)/3
5867 reflections	(Δ/σ)_max_ = 0.002
271 parameters	Δρ_max_ = 0.26 e Å^−^^3^
0 restraints	Δρ_min_ = −0.24 e Å^−^^3^

### Special details

**Table:**

Experimental. CrysAlis RED, Oxford Diffraction Ltd., Version 1.171.33.32 (release 27-01-2009 CrysAlis171 .NET) (compiled Jan 27 2009,14:17:37) Empirical absorption correction using spherical harmonics, implemented in SCALE3 ABSPACK scaling algorithm.
Geometry. All e.s.d.'s (except the e.s.d. in the dihedral angle between two l.s. planes) are estimated using the full covariance matrix. The cell e.s.d.'s are taken into account individually in the estimation of e.s.d.'s in distances, angles and torsion angles; correlations between e.s.d.'s in cell parameters are only used when they are defined by crystal symmetry. An approximate (isotropic) treatment of cell e.s.d.'s is used for estimating e.s.d.'s involving l.s. planes.
Refinement. Refinement of *F*^2^ against ALL reflections. The weighted *R*-factor *wR* and goodness of fit *S* are based on *F*^2^, conventional *R*-factors *R* are based on *F*, with *F* set to zero for negative *F*^2^. The threshold expression of *F*^2^ > σ(*F*^2^) is used only for calculating *R*-factors(gt) *etc*. and is not relevant to the choice of reflections for refinement. *R*-factors based on *F*^2^ are statistically about twice as large as those based on *F*, and *R*- factors based on ALL data will be even larger.

### Fractional atomic coordinates and isotropic or equivalent isotropic displacement parameters (Å^2^)

**Table:**

	*x*	*y*	*z*	*U*_iso_*/*U*_eq_	
Fe1	0.22827 (2)	0.555286 (18)	0.788568 (11)	0.04543 (7)	
N1	0.31428 (12)	0.20402 (10)	0.81870 (6)	0.0418 (3)	
O1	0.08238 (10)	0.25999 (10)	0.72376 (6)	0.0561 (3)	
O2	0.32130 (12)	0.17948 (11)	0.93657 (6)	0.0685 (3)	
O3	0.35548 (15)	0.01217 (10)	0.80453 (6)	0.0727 (4)	
C1	0.22015 (15)	0.39344 (13)	0.83023 (7)	0.0435 (3)	
C2	0.35480 (17)	0.44935 (13)	0.88449 (8)	0.0580 (4)	
H2	0.4467	0.4217	0.9000	0.070*	
C3	0.3229 (2)	0.55509 (15)	0.91043 (9)	0.0733 (5)	
H3	0.3911	0.6086	0.9468	0.088*	
C4	0.1724 (2)	0.56619 (15)	0.87266 (9)	0.0668 (5)	
H4	0.1238	0.6281	0.8795	0.080*	
C5	0.10714 (17)	0.46776 (13)	0.82268 (9)	0.0524 (4)	
H5	0.0079	0.4534	0.7904	0.063*	
C6	0.18399 (19)	0.54441 (15)	0.67438 (9)	0.0612 (4)	
H6	0.1552	0.4768	0.6427	0.073*	
C7	0.3267 (2)	0.58116 (17)	0.72643 (11)	0.0699 (5)	
H7	0.4100	0.5425	0.7361	0.084*	
C8	0.3216 (2)	0.68789 (18)	0.76171 (12)	0.0761 (5)	
H8	0.4012	0.7328	0.7986	0.091*	
C9	0.1762 (2)	0.71431 (16)	0.73166 (11)	0.0713 (5)	
H9	0.1419	0.7796	0.7453	0.086*	
C10	0.09164 (19)	0.62606 (17)	0.67781 (10)	0.0674 (5)	
H10	−0.0093	0.6222	0.6490	0.081*	
C11	0.19470 (14)	0.28448 (13)	0.78516 (7)	0.0414 (3)	
C12	0.38958 (17)	0.18287 (12)	0.90480 (8)	0.0483 (4)	
C13	0.54864 (16)	0.17184 (13)	0.94748 (8)	0.0498 (4)	
C14	0.62677 (17)	0.21187 (15)	0.91517 (9)	0.0612 (4)	
H14	0.5780	0.2427	0.8633	0.073*	
C15	0.77638 (18)	0.20666 (18)	0.95886 (11)	0.0778 (6)	
H15	0.8286	0.2345	0.9370	0.093*	
C16	0.8472 (2)	0.1599 (2)	1.03492 (13)	0.0939 (7)	
H16	0.9481	0.1558	1.0647	0.113*	
C17	0.7712 (3)	0.11922 (18)	1.06751 (11)	0.0896 (7)	
H17	0.8208	0.0871	1.1190	0.107*	
C18	0.6221 (2)	0.12535 (14)	1.02499 (8)	0.0666 (5)	
H18	0.5709	0.0987	1.0477	0.080*	
C19	0.31625 (16)	0.10751 (14)	0.77336 (8)	0.0475 (3)	
C20	0.27699 (15)	0.13020 (14)	0.68970 (8)	0.0476 (4)	
C21	0.29771 (16)	0.23823 (15)	0.66520 (8)	0.0555 (4)	
H21	0.3308	0.3018	0.7006	0.067*	
C22	0.2693 (2)	0.25240 (18)	0.58799 (10)	0.0744 (5)	
H22	0.2849	0.3250	0.5717	0.089*	
C23	0.2179 (2)	0.1583 (2)	0.53545 (11)	0.0884 (6)	
H23	0.1969	0.1679	0.4832	0.106*	
C24	0.1979 (2)	0.0515 (2)	0.55973 (11)	0.0918 (7)	
H24	0.1638	−0.0116	0.5240	0.110*	
C25	0.2275 (2)	0.03562 (17)	0.63671 (9)	0.0700 (5)	
H25	0.2144	−0.0379	0.6530	0.084*	

### Atomic displacement parameters (Å^2^)

**Table:**

	*U*^11^	*U*^22^	*U*^33^	*U*^12^	*U*^13^	*U*^23^
Fe1	0.04328 (12)	0.04295 (12)	0.04387 (11)	0.00224 (11)	0.01901 (9)	0.00300 (9)
N1	0.0486 (7)	0.0418 (7)	0.0316 (5)	0.0062 (6)	0.0190 (5)	0.0003 (5)
O1	0.0399 (5)	0.0705 (7)	0.0479 (6)	−0.0040 (5)	0.0166 (5)	−0.0101 (5)
O2	0.0894 (8)	0.0766 (8)	0.0531 (6)	0.0206 (7)	0.0471 (6)	0.0179 (6)
O3	0.1159 (10)	0.0427 (6)	0.0520 (6)	0.0101 (7)	0.0396 (7)	−0.0003 (5)
C1	0.0496 (8)	0.0431 (8)	0.0348 (6)	0.0072 (7)	0.0205 (6)	0.0042 (6)
C2	0.0570 (9)	0.0497 (9)	0.0400 (7)	0.0107 (8)	0.0073 (7)	0.0004 (7)
C3	0.0999 (14)	0.0470 (10)	0.0387 (8)	0.0114 (10)	0.0137 (9)	−0.0047 (7)
C4	0.1046 (15)	0.0523 (10)	0.0520 (9)	0.0247 (10)	0.0474 (10)	0.0090 (8)
C5	0.0644 (10)	0.0530 (10)	0.0513 (8)	0.0125 (8)	0.0385 (8)	0.0119 (7)
C6	0.0733 (11)	0.0618 (11)	0.0518 (9)	−0.0044 (10)	0.0354 (9)	0.0059 (8)
C7	0.0639 (11)	0.0749 (14)	0.0875 (12)	0.0072 (10)	0.0515 (10)	0.0173 (10)
C8	0.0698 (12)	0.0665 (13)	0.0913 (13)	−0.0222 (10)	0.0424 (11)	−0.0031 (11)
C9	0.0925 (14)	0.0491 (10)	0.0868 (13)	0.0111 (11)	0.0574 (12)	0.0188 (10)
C10	0.0572 (10)	0.0787 (13)	0.0605 (10)	0.0082 (10)	0.0275 (8)	0.0297 (10)
C11	0.0436 (8)	0.0492 (9)	0.0349 (7)	0.0033 (7)	0.0234 (6)	0.0060 (6)
C12	0.0696 (10)	0.0371 (8)	0.0360 (7)	0.0094 (8)	0.0265 (7)	0.0026 (6)
C13	0.0624 (9)	0.0385 (8)	0.0348 (7)	0.0101 (8)	0.0166 (7)	−0.0026 (6)
C14	0.0598 (10)	0.0677 (11)	0.0431 (8)	0.0110 (9)	0.0187 (8)	−0.0091 (8)
C15	0.0601 (11)	0.0908 (14)	0.0669 (11)	0.0058 (11)	0.0233 (9)	−0.0230 (10)
C16	0.0661 (12)	0.0903 (16)	0.0712 (13)	0.0221 (12)	0.0000 (11)	−0.0230 (12)
C17	0.0973 (16)	0.0644 (13)	0.0459 (10)	0.0186 (13)	−0.0031 (11)	−0.0004 (9)
C18	0.0876 (13)	0.0460 (10)	0.0382 (8)	0.0067 (9)	0.0145 (8)	0.0002 (7)
C19	0.0541 (9)	0.0447 (9)	0.0412 (7)	0.0012 (8)	0.0237 (7)	−0.0046 (7)
C20	0.0484 (8)	0.0552 (10)	0.0418 (7)	0.0021 (7)	0.0257 (7)	−0.0038 (7)
C21	0.0600 (9)	0.0643 (11)	0.0493 (8)	0.0031 (8)	0.0339 (8)	−0.0016 (8)
C22	0.0892 (13)	0.0874 (14)	0.0646 (11)	0.0029 (12)	0.0530 (10)	0.0117 (10)
C23	0.1114 (16)	0.1143 (19)	0.0530 (10)	−0.0061 (15)	0.0529 (11)	−0.0038 (12)
C24	0.1254 (18)	0.1016 (17)	0.0601 (11)	−0.0254 (14)	0.0571 (12)	−0.0279 (11)
C25	0.0933 (13)	0.0693 (12)	0.0548 (9)	−0.0129 (10)	0.0444 (10)	−0.0146 (8)

### Geometric parameters (Å, °)

**Table:**

Fe1—C5	2.0260 (15)	C7—H7	0.9300
Fe1—C7	2.0269 (17)	C8—C9	1.394 (2)
Fe1—C6	2.0303 (15)	C8—H8	0.9300
Fe1—C1	2.0300 (14)	C9—C10	1.389 (2)
Fe1—C2	2.0309 (14)	C9—H9	0.9300
Fe1—C8	2.0339 (18)	C10—H10	0.9300
Fe1—C10	2.0349 (15)	C12—C13	1.472 (2)
Fe1—C9	2.0363 (16)	C13—C14	1.379 (2)
Fe1—C3	2.0399 (15)	C13—C18	1.3920 (19)
Fe1—C4	2.0430 (16)	C14—C15	1.378 (2)
N1—C19	1.4177 (17)	C14—H14	0.9300
N1—C11	1.4300 (17)	C15—C16	1.371 (3)
N1—C12	1.4531 (16)	C15—H15	0.9300
O1—C11	1.2061 (15)	C16—C17	1.367 (3)
O2—C12	1.1973 (16)	C16—H16	0.9300
O3—C19	1.2030 (17)	C17—C18	1.374 (3)
C1—C2	1.421 (2)	C17—H17	0.9300
C1—C5	1.431 (2)	C18—H18	0.9300
C1—C11	1.458 (2)	C19—C20	1.4830 (19)
C2—C3	1.416 (2)	C20—C21	1.377 (2)
C2—H2	0.9300	C20—C25	1.388 (2)
C3—C4	1.396 (3)	C21—C22	1.386 (2)
C3—H3	0.9300	C21—H21	0.9300
C4—C5	1.404 (2)	C22—C23	1.379 (3)
C4—H4	0.9300	C22—H22	0.9300
C5—H5	0.9300	C23—C24	1.361 (3)
C6—C7	1.392 (2)	C23—H23	0.9300
C6—C10	1.393 (2)	C24—C25	1.379 (2)
C6—H6	0.9300	C24—H24	0.9300
C7—C8	1.410 (2)	C25—H25	0.9300
			
C5—Fe1—C7	156.04 (7)	Fe1—C5—H5	125.6
C5—Fe1—C6	121.39 (7)	C7—C6—C10	108.43 (16)
C7—Fe1—C6	40.14 (7)	C7—C6—Fe1	69.80 (9)
C5—Fe1—C1	41.32 (6)	C10—C6—Fe1	70.14 (9)
C7—Fe1—C1	121.55 (7)	C7—C6—H6	125.8
C6—Fe1—C1	110.74 (6)	C10—C6—H6	125.8
C5—Fe1—C2	69.06 (7)	Fe1—C6—H6	125.9
C7—Fe1—C2	109.23 (7)	C6—C7—C8	107.29 (16)
C6—Fe1—C2	129.30 (7)	C6—C7—Fe1	70.06 (9)
C1—Fe1—C2	40.98 (6)	C8—C7—Fe1	69.95 (10)
C5—Fe1—C8	161.66 (7)	C6—C7—H7	126.4
C7—Fe1—C8	40.64 (7)	C8—C7—H7	126.4
C6—Fe1—C8	67.47 (7)	Fe1—C7—H7	125.2
C1—Fe1—C8	155.13 (7)	C9—C8—C7	107.99 (17)
C2—Fe1—C8	119.49 (8)	C9—C8—Fe1	70.07 (10)
C5—Fe1—C10	108.23 (6)	C7—C8—Fe1	69.42 (10)
C7—Fe1—C10	67.61 (7)	C9—C8—H8	126.0
C6—Fe1—C10	40.09 (7)	C7—C8—H8	126.0
C1—Fe1—C10	128.56 (7)	Fe1—C8—H8	126.1
C2—Fe1—C10	166.69 (7)	C10—C9—C8	108.05 (17)
C8—Fe1—C10	67.22 (7)	C10—C9—Fe1	69.99 (10)
C5—Fe1—C9	125.12 (7)	C8—C9—Fe1	69.88 (10)
C7—Fe1—C9	67.87 (7)	C10—C9—H9	126.0
C6—Fe1—C9	67.34 (7)	C8—C9—H9	126.0
C1—Fe1—C9	164.26 (7)	Fe1—C9—H9	125.7
C2—Fe1—C9	152.35 (8)	C9—C10—C6	108.24 (16)
C8—Fe1—C9	40.05 (7)	C9—C10—Fe1	70.10 (9)
C10—Fe1—C9	39.91 (7)	C6—C10—Fe1	69.78 (9)
C5—Fe1—C3	67.84 (7)	C9—C10—H10	125.9
C7—Fe1—C3	127.41 (8)	C6—C10—H10	125.9
C6—Fe1—C3	165.89 (8)	Fe1—C10—H10	125.8
C1—Fe1—C3	68.26 (6)	O1—C11—N1	120.15 (13)
C2—Fe1—C3	40.71 (6)	O1—C11—C1	124.46 (13)
C8—Fe1—C3	107.15 (8)	N1—C11—C1	115.38 (12)
C10—Fe1—C3	151.55 (8)	O2—C12—N1	119.28 (14)
C9—Fe1—C3	117.63 (8)	O2—C12—C13	124.53 (13)
C5—Fe1—C4	40.37 (6)	N1—C12—C13	116.14 (13)
C7—Fe1—C4	163.00 (8)	C14—C13—C18	119.43 (16)
C6—Fe1—C4	153.85 (8)	C14—C13—C12	122.32 (13)
C1—Fe1—C4	68.54 (6)	C18—C13—C12	118.14 (16)
C2—Fe1—C4	68.45 (7)	C15—C14—C13	120.74 (16)
C8—Fe1—C4	124.62 (8)	C15—C14—H14	119.6
C10—Fe1—C4	118.52 (7)	C13—C14—H14	119.6
C9—Fe1—C4	105.92 (7)	C16—C15—C14	119.2 (2)
C3—Fe1—C4	40.00 (7)	C16—C15—H15	120.4
C19—N1—C11	121.13 (11)	C14—C15—H15	120.4
C19—N1—C12	114.53 (11)	C17—C16—C15	120.73 (19)
C11—N1—C12	116.91 (11)	C17—C16—H16	119.6
C2—C1—C5	107.47 (13)	C15—C16—H16	119.6
C2—C1—C11	128.32 (13)	C16—C17—C18	120.67 (17)
C5—C1—C11	124.12 (13)	C16—C17—H17	119.7
C2—C1—Fe1	69.54 (8)	C18—C17—H17	119.7
C5—C1—Fe1	69.19 (8)	C17—C18—C13	119.24 (19)
C11—C1—Fe1	123.82 (9)	C17—C18—H18	120.4
C3—C2—C1	107.17 (15)	C13—C18—H18	120.4
C3—C2—Fe1	69.99 (8)	O3—C19—N1	119.61 (12)
C1—C2—Fe1	69.48 (8)	O3—C19—C20	122.30 (13)
C3—C2—H2	126.4	N1—C19—C20	118.01 (13)
C1—C2—H2	126.4	C21—C20—C25	119.73 (14)
Fe1—C2—H2	125.7	C21—C20—C19	122.44 (13)
C4—C3—C2	109.11 (15)	C25—C20—C19	117.66 (14)
C4—C3—Fe1	70.12 (8)	C20—C21—C22	120.14 (16)
C2—C3—Fe1	69.30 (8)	C20—C21—H21	119.9
C4—C3—H3	125.4	C22—C21—H21	119.9
C2—C3—H3	125.4	C23—C22—C21	119.62 (18)
Fe1—C3—H3	126.7	C23—C22—H22	120.2
C3—C4—C5	108.25 (15)	C21—C22—H22	120.2
C3—C4—Fe1	69.88 (10)	C24—C23—C22	120.22 (17)
C5—C4—Fe1	69.17 (9)	C24—C23—H23	119.9
C3—C4—H4	125.9	C22—C23—H23	119.9
C5—C4—H4	125.9	C23—C24—C25	120.80 (18)
Fe1—C4—H4	126.6	C23—C24—H24	119.6
C4—C5—C1	107.99 (15)	C25—C24—H24	119.6
C4—C5—Fe1	70.47 (10)	C24—C25—C20	119.47 (18)
C1—C5—Fe1	69.49 (8)	C24—C25—H25	120.3
C4—C5—H5	126.0	C20—C25—H25	120.3
C1—C5—H5	126.0		
			
C5—Fe1—C1—C2	−118.97 (12)	C4—Fe1—C6—C10	42.5 (2)
C7—Fe1—C1—C2	83.25 (11)	C10—C6—C7—C8	−0.62 (19)
C6—Fe1—C1—C2	126.64 (10)	Fe1—C6—C7—C8	−60.33 (12)
C8—Fe1—C1—C2	45.68 (19)	C10—C6—C7—Fe1	59.71 (11)
C10—Fe1—C1—C2	168.54 (10)	C5—Fe1—C7—C6	47.3 (2)
C9—Fe1—C1—C2	−153.4 (2)	C1—Fe1—C7—C6	85.21 (12)
C3—Fe1—C1—C2	−38.25 (10)	C2—Fe1—C7—C6	128.82 (10)
C4—Fe1—C1—C2	−81.42 (10)	C8—Fe1—C7—C6	−117.97 (16)
C7—Fe1—C1—C5	−157.78 (10)	C10—Fe1—C7—C6	−37.35 (10)
C6—Fe1—C1—C5	−114.38 (9)	C9—Fe1—C7—C6	−80.62 (12)
C2—Fe1—C1—C5	118.97 (12)	C3—Fe1—C7—C6	170.76 (10)
C8—Fe1—C1—C5	164.66 (15)	C4—Fe1—C7—C6	−152.1 (2)
C10—Fe1—C1—C5	−72.49 (11)	C5—Fe1—C7—C8	165.24 (15)
C9—Fe1—C1—C5	−34.5 (3)	C6—Fe1—C7—C8	117.97 (16)
C3—Fe1—C1—C5	80.72 (10)	C1—Fe1—C7—C8	−156.81 (10)
C4—Fe1—C1—C5	37.56 (9)	C2—Fe1—C7—C8	−113.21 (11)
C5—Fe1—C1—C11	117.88 (15)	C10—Fe1—C7—C8	80.63 (12)
C7—Fe1—C1—C11	−39.89 (15)	C9—Fe1—C7—C8	37.35 (11)
C6—Fe1—C1—C11	3.50 (14)	C3—Fe1—C7—C8	−71.27 (13)
C2—Fe1—C1—C11	−123.14 (16)	C4—Fe1—C7—C8	−34.1 (3)
C8—Fe1—C1—C11	−77.5 (2)	C6—C7—C8—C9	0.7 (2)
C10—Fe1—C1—C11	45.39 (15)	Fe1—C7—C8—C9	−59.68 (12)
C9—Fe1—C1—C11	83.4 (3)	C6—C7—C8—Fe1	60.40 (11)
C3—Fe1—C1—C11	−161.40 (14)	C5—Fe1—C8—C9	−41.7 (3)
C4—Fe1—C1—C11	155.44 (14)	C7—Fe1—C8—C9	119.15 (17)
C5—C1—C2—C3	1.11 (16)	C6—Fe1—C8—C9	81.09 (12)
C11—C1—C2—C3	177.67 (14)	C1—Fe1—C8—C9	172.06 (13)
Fe1—C1—C2—C3	60.12 (11)	C2—Fe1—C8—C9	−155.32 (11)
C5—C1—C2—Fe1	−59.02 (9)	C10—Fe1—C8—C9	37.49 (11)
C11—C1—C2—Fe1	117.55 (14)	C3—Fe1—C8—C9	−112.78 (12)
C5—Fe1—C2—C3	−79.95 (12)	C4—Fe1—C8—C9	−72.35 (14)
C7—Fe1—C2—C3	125.52 (12)	C5—Fe1—C8—C7	−160.81 (19)
C6—Fe1—C2—C3	165.99 (11)	C6—Fe1—C8—C7	−38.06 (11)
C1—Fe1—C2—C3	−118.15 (15)	C1—Fe1—C8—C7	52.9 (2)
C8—Fe1—C2—C3	82.07 (14)	C2—Fe1—C8—C7	85.53 (12)
C10—Fe1—C2—C3	−160.6 (3)	C10—Fe1—C8—C7	−81.65 (12)
C9—Fe1—C2—C3	46.7 (2)	C9—Fe1—C8—C7	−119.15 (17)
C4—Fe1—C2—C3	−36.51 (12)	C3—Fe1—C8—C7	128.07 (11)
C5—Fe1—C2—C1	38.20 (9)	C4—Fe1—C8—C7	168.50 (11)
C7—Fe1—C2—C1	−116.33 (10)	C7—C8—C9—C10	−0.6 (2)
C6—Fe1—C2—C1	−75.85 (12)	Fe1—C8—C9—C10	−59.83 (12)
C8—Fe1—C2—C1	−159.77 (9)	C7—C8—C9—Fe1	59.27 (12)
C10—Fe1—C2—C1	−42.5 (3)	C5—Fe1—C9—C10	−75.83 (13)
C9—Fe1—C2—C1	164.85 (14)	C7—Fe1—C9—C10	81.10 (12)
C3—Fe1—C2—C1	118.15 (15)	C6—Fe1—C9—C10	37.52 (10)
C4—Fe1—C2—C1	81.65 (10)	C1—Fe1—C9—C10	−48.7 (3)
C1—C2—C3—C4	−0.84 (18)	C2—Fe1—C9—C10	170.52 (14)
Fe1—C2—C3—C4	58.96 (11)	C8—Fe1—C9—C10	118.98 (17)
C1—C2—C3—Fe1	−59.80 (10)	C3—Fe1—C9—C10	−157.08 (12)
C5—Fe1—C3—C4	−37.38 (10)	C4—Fe1—C9—C10	−115.66 (12)
C7—Fe1—C3—C4	164.07 (11)	C5—Fe1—C9—C8	165.18 (11)
C6—Fe1—C3—C4	−170.8 (3)	C7—Fe1—C9—C8	−37.88 (11)
C1—Fe1—C3—C4	−82.09 (11)	C6—Fe1—C9—C8	−81.46 (12)
C2—Fe1—C3—C4	−120.59 (16)	C1—Fe1—C9—C8	−167.6 (2)
C8—Fe1—C3—C4	123.87 (11)	C2—Fe1—C9—C8	51.5 (2)
C10—Fe1—C3—C4	50.2 (2)	C10—Fe1—C9—C8	−118.98 (17)
C9—Fe1—C3—C4	81.82 (12)	C3—Fe1—C9—C8	83.94 (14)
C5—Fe1—C3—C2	83.21 (11)	C4—Fe1—C9—C8	125.36 (12)
C7—Fe1—C3—C2	−75.34 (14)	C8—C9—C10—C6	0.17 (19)
C6—Fe1—C3—C2	−50.2 (3)	Fe1—C9—C10—C6	−59.58 (11)
C1—Fe1—C3—C2	38.50 (10)	C8—C9—C10—Fe1	59.76 (12)
C8—Fe1—C3—C2	−115.55 (12)	C7—C6—C10—C9	0.28 (18)
C10—Fe1—C3—C2	170.78 (14)	Fe1—C6—C10—C9	59.78 (11)
C9—Fe1—C3—C2	−157.59 (11)	C7—C6—C10—Fe1	−59.50 (11)
C4—Fe1—C3—C2	120.59 (16)	C5—Fe1—C10—C9	123.38 (11)
C2—C3—C4—C5	0.24 (18)	C7—Fe1—C10—C9	−81.81 (12)
Fe1—C3—C4—C5	58.70 (10)	C6—Fe1—C10—C9	−119.21 (15)
C2—C3—C4—Fe1	−58.46 (11)	C1—Fe1—C10—C9	164.91 (10)
C5—Fe1—C4—C3	119.75 (14)	C2—Fe1—C10—C9	−160.6 (3)
C7—Fe1—C4—C3	−48.2 (3)	C8—Fe1—C10—C9	−37.63 (11)
C6—Fe1—C4—C3	174.93 (14)	C3—Fe1—C10—C9	46.4 (2)
C1—Fe1—C4—C3	81.33 (10)	C4—Fe1—C10—C9	80.61 (13)
C2—Fe1—C4—C3	37.14 (10)	C5—Fe1—C10—C6	−117.41 (10)
C8—Fe1—C4—C3	−74.61 (12)	C7—Fe1—C10—C6	37.40 (10)
C10—Fe1—C4—C3	−155.39 (10)	C1—Fe1—C10—C6	−75.88 (12)
C9—Fe1—C4—C3	−114.23 (11)	C2—Fe1—C10—C6	−41.4 (3)
C7—Fe1—C4—C5	−168.0 (2)	C8—Fe1—C10—C6	81.58 (11)
C6—Fe1—C4—C5	55.18 (19)	C9—Fe1—C10—C6	119.21 (15)
C1—Fe1—C4—C5	−38.42 (9)	C3—Fe1—C10—C6	165.63 (15)
C2—Fe1—C4—C5	−82.61 (10)	C4—Fe1—C10—C6	−160.18 (10)
C8—Fe1—C4—C5	165.64 (10)	C19—N1—C11—O1	13.67 (19)
C10—Fe1—C4—C5	84.86 (11)	C12—N1—C11—O1	−134.58 (14)
C9—Fe1—C4—C5	126.02 (10)	C19—N1—C11—C1	−166.52 (12)
C3—Fe1—C4—C5	−119.75 (14)	C12—N1—C11—C1	45.23 (16)
C3—C4—C5—C1	0.46 (17)	C2—C1—C11—O1	−156.27 (14)
Fe1—C4—C5—C1	59.60 (10)	C5—C1—C11—O1	19.8 (2)
C3—C4—C5—Fe1	−59.14 (12)	Fe1—C1—C11—O1	−66.65 (18)
C2—C1—C5—C4	−0.98 (16)	C2—C1—C11—N1	23.9 (2)
C11—C1—C5—C4	−177.72 (13)	C5—C1—C11—N1	−160.03 (12)
Fe1—C1—C5—C4	−60.21 (10)	Fe1—C1—C11—N1	113.55 (11)
C2—C1—C5—Fe1	59.24 (10)	C19—N1—C12—O2	−113.74 (16)
C11—C1—C5—Fe1	−117.51 (12)	C11—N1—C12—O2	36.58 (19)
C7—Fe1—C5—C4	171.38 (16)	C19—N1—C12—C13	68.71 (16)
C6—Fe1—C5—C4	−154.92 (10)	C11—N1—C12—C13	−140.97 (13)
C1—Fe1—C5—C4	118.85 (13)	O2—C12—C13—C14	−159.20 (16)
C2—Fe1—C5—C4	80.96 (10)	N1—C12—C13—C14	18.2 (2)
C8—Fe1—C5—C4	−40.4 (3)	O2—C12—C13—C18	17.1 (2)
C10—Fe1—C5—C4	−112.88 (11)	N1—C12—C13—C18	−165.53 (12)
C9—Fe1—C5—C4	−71.97 (12)	C18—C13—C14—C15	−0.2 (2)
C3—Fe1—C5—C4	37.05 (9)	C12—C13—C14—C15	175.99 (15)
C7—Fe1—C5—C1	52.53 (19)	C13—C14—C15—C16	0.7 (3)
C6—Fe1—C5—C1	86.23 (10)	C14—C15—C16—C17	−0.2 (3)
C2—Fe1—C5—C1	−37.90 (8)	C15—C16—C17—C18	−0.6 (3)
C8—Fe1—C5—C1	−159.3 (2)	C16—C17—C18—C13	1.1 (3)
C10—Fe1—C5—C1	128.27 (9)	C14—C13—C18—C17	−0.6 (2)
C9—Fe1—C5—C1	169.18 (9)	C12—C13—C18—C17	−177.01 (15)
C3—Fe1—C5—C1	−81.80 (9)	C11—N1—C19—O3	−136.69 (15)
C4—Fe1—C5—C1	−118.85 (13)	C12—N1—C19—O3	12.3 (2)
C5—Fe1—C6—C7	−159.55 (10)	C11—N1—C19—C20	46.64 (18)
C1—Fe1—C6—C7	−114.76 (11)	C12—N1—C19—C20	−164.41 (13)
C2—Fe1—C6—C7	−71.92 (13)	O3—C19—C20—C21	−148.83 (16)
C8—Fe1—C6—C7	38.52 (11)	N1—C19—C20—C21	27.7 (2)
C10—Fe1—C6—C7	119.42 (15)	O3—C19—C20—C25	26.5 (2)
C9—Fe1—C6—C7	82.06 (12)	N1—C19—C20—C25	−156.95 (14)
C3—Fe1—C6—C7	−31.6 (3)	C25—C20—C21—C22	0.0 (2)
C4—Fe1—C6—C7	161.93 (15)	C19—C20—C21—C22	175.18 (15)
C5—Fe1—C6—C10	81.04 (12)	C20—C21—C22—C23	1.1 (3)
C7—Fe1—C6—C10	−119.42 (15)	C21—C22—C23—C24	−1.3 (3)
C1—Fe1—C6—C10	125.82 (10)	C22—C23—C24—C25	0.4 (3)
C2—Fe1—C6—C10	168.66 (10)	C23—C24—C25—C20	0.6 (3)
C8—Fe1—C6—C10	−80.90 (11)	C21—C20—C25—C24	−0.8 (3)
C9—Fe1—C6—C10	−37.36 (10)	C19—C20—C25—C24	−176.26 (17)
C3—Fe1—C6—C10	−151.0 (3)		

### Hydrogen-bond geometry (Å, °)

**Table:**

Cg2 is the centroid of the C6–C10 ring.

**Table:**

*D*—H···*A*	*D*—H	H···*A*	*D*···*A*	*D*—H···*A*
C4—H4···O1^i^	0.93	2.56	3.241 (2)	131
C15—H15···Cg2^ii^	0.93	2.97	3.528 (2)	120

Symmetry codes: (i) −*x*, *y*+1/2, −*z*+3/2; (ii) −*x*+1, *y*−1/2, −*z*+3/2.
